# BALROG-MON: a high-throughput pipeline for Bacterial AntimicrobiaL Resistance annOtation of Genomes-Metagenomic Oxford Nanopore

**DOI:** 10.17912/micropub.biology.001427

**Published:** 2025-02-19

**Authors:** Edward Bird, Victoria Pickens, David Molik, Kristopher Silver, Dana Nayduch

**Affiliations:** 1 Entomology, Kansas State University, Manhattan, Kansas, United States; 2 Arthropod-Borne Animal Diseases Research Unit, Agricultural Research Service, United States Department of Agriculture, Manhattan, KS, United States

## Abstract

BALROG-MON is a Nextflow pipeline for automated analysis of metagenomic long-read data to detect pathogens, annotate antimicrobial resistance genes (ARGs), link ARGs to specific pathogens, predict ARG origin (e.g., plasmid, chromosomal) and optionally perform steps like community analysis. With both assembly-based and assembly-free workflows, BALROG-MON is applicable to a wide range of sample types with low or high coverage, varying complexities and origins. Optional genome binning provides a comprehensive overview of ARGs within the dataset. BALROG-MON additionally presents results in summarized reports, overall serving as a flexible analysis tool for exploring diverse metagenomic samples for pathogens and antibiotic resistance.

**Figure 1. BALROG-MON workflow diagram.**
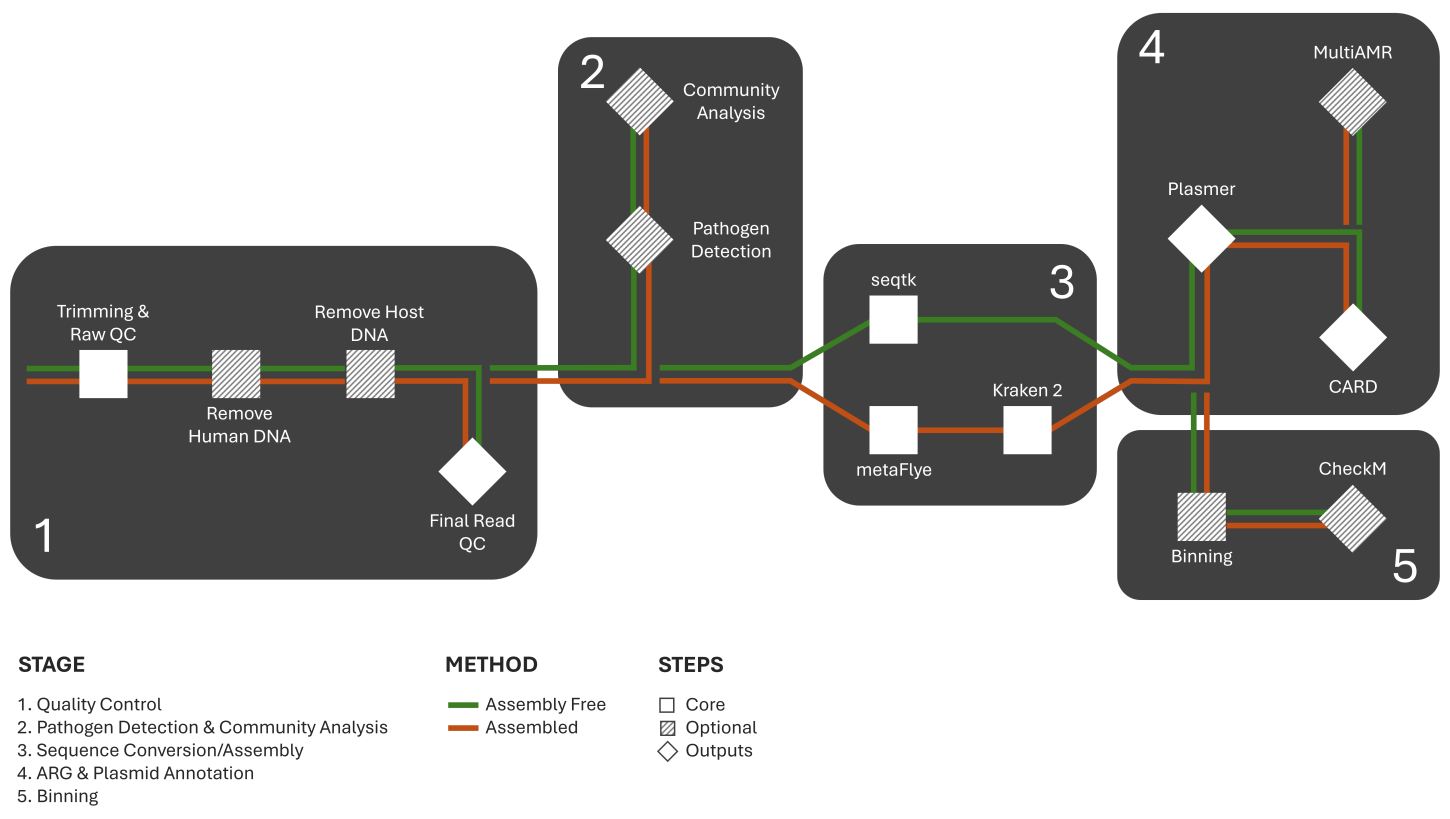
BALROG-MON enables the option of using an “assembled” or “assembly-free” method for the annotation of ARGs from metagenomic long read sequences.

## Description

BALROG-MON (Bacterial AntimicrobiaL Resistance annOtation of Genomes – Metagenomic Oxford Nanopore) ( https://github.com/edwardbirdlab/BALROG-MON/ ) automates the analysis of antimicrobial resistance genes (ARGs) from complex metagenomes. Antimicrobial resistance (AMR) is a significant global health challenge exacerbated by the increasing prevalence of resistance to and the declining discovery of new antibiotics. Over the past decade, multiple bioinformatic tools and databases have been developed to identify, characterize and understand the evolution of ARGs. While many tools focus on isolated bacterial genomes, BALROG-MON analyzes antimicrobial resistance metagenomically, offering a high-throughput approach to study ARGs across entire environments. It also provides insights into AMR in pathogens without the need for culture-based methods, making it a powerful tool for understanding resistance in complex microbial communities and what threats may be present.

Metagenomic sequencing is a recently feasible method that can generate data from which ARGs, microbiomes, and pathogens in the sample can be characterized simultaneously, serving as a more effective and comprehensive method than isolating and culturing bacteria. Typical analysis of metagenomic data involves either an assembly-based approach or a read-based approach, with each having its own benefits and limitations. Metagenomic assembly allows for upstream or downstream investigation of ARGs and provides accurate identification of their origin. However, this approach may lead to information loss, as low-coverage genomes are often not assembled. In contrast, read-based approaches enable mapping of all available data but lack the capability to explore surrounding genomic context or provide accurate taxonomic classifications. To address these challenges, we developed BALROG-MON, a versatile and reproducible Nextflow pipeline for surveying pathogens and ARGs from metagenomic long-read sequencing, offering both “assembled” and “assembly-free” workflow options.

BALROG-MON v1.0 ( DOI: 10.5281/zenodo.14850876 ) consists of six major steps: (1) Quality Control, (2) Pathogen Detection and Community Analysis, (3) Sequence Conversion/Assembly, (4) ARG and Plasmid Annotation, (5) Binning ( *optional* ) and (6) Output Collection and Summary. Written in Nextflow, BALROG-MON compartmentalizes each process, allowing users to easily modify or customize steps to suit their analysis needs. All processes are executed with a single command, simplifying the workflow while ensuring scalability. Docker containers manage all dependencies, ensuring reproducibility across different computing environments. During data quality control host sequences and human sequences can be removed, and low-quality bases and adapters are trimmed. Quality-controlled sequences are then either assembled in assembly mode or converted to FASTA format in assembly-free mode. For high-coverage metagenomes, binning can be enabled, allowing reads or contigs to be grouped for generating metagenomically reconstructed genomes. The resulting sequences are classified as either plasmid or chromosomal in origin. ARGs are annotated using multiple tools, and the outputs are standardized. ARGs, taxonomic classifications, and plasmid classification results are integrated to generate a comprehensive report for each sample. Additionally, all quality control metrics (trimming, host and human sequence removal, final read QC) are summarized in a final report.

Input data and, if also provided, reference genome(s) are first run through data_validator v1.0 (https://github.com/edwardbirdlab/nextflow_input_std), a custom Python script that checks input data for a valid format and, if necessary, reformats FASTQ and FASTA files to the expected format. FastQC v0.12.1 (https://www.bioinformatics.babraham.ac.uk/projects/fastqc/) is run on raw reads to provide statistics on the quality of sequencing, and Porechop v0.2.4 (https://github.com/rrwick/Porechop) and chopper v0.7.0 (https://github.com/wdecoster/chopper) quality control reads. Optionally, reads can be mapped to the human genome (GCA_000001405.15_GRCh38) using minimap2 v2.26 (https://github.com/lh3/minimap2), and then non-human reads extracted using Samtools v1.17 (https://www.htslib.org/). Reads can also be mapped to one or more provided reference genomes, and host sequences removed as described above. Final quality-controlled host- and/or human-depleted reads are run through FastqQC v0.12.1 for final quality metrics.

Quality-controlled reads are classified utilizing Kraken 2 v2.1.3 (https://github.com/DerrickWood/kraken2) and Kraken’s pre-built PlusPFP ( https://benlangmead.github.io/aws-indexes/k2 ) database by default. Optionally, species-level composition of the metagenome can be estimated using Bracken v2.9 (https://github.com/jenniferlu717/Bracken) and the same database used in Kraken 2 sequence identification. KrakenTools v1.2 (https://github.com/jenniferlu717/KrakenTools) then calculates Shannon’s alpha diversity and Bray-Curtis dissimilarity (Beta Diversity), as well as a Krona chart and MPA style report.

The sequence processing module workflow differs between BALROG-MON run modes (e.g., “assembly” or “assembly-free”). In assembly-free mode, quality-controlled reads are simply converted from FASTQ to FASTA format with SeqTK v1.4 (https://github.com/lh3/seqtk). In assembly mode, a draft metagenome will be assembled with metaFlye v2.9.3 (https://github.com/mikolmogorov/Flye) and contigs re-identified with Kraken 2 and the PlusPFP database. Finally, in both assembly or assembly-free mode, QUAST v5.2.0 (https://github.com/ablab/quast) is run to gather statistics (N50, Total Length, etc.) on the output sequences.

Sequences are first predicted to be of plasmid or chromosomal origin using Plasmer v23.04.20 (https://github.com/nekokoe/Plasmer), and then renamed to reflect their origin. ARGs are then annotated using Resistance Gene Identifier (RGI) v6.0.3 (https://github.com/arpcard/rgi) and the Comprehensive Antibiotic Resistance Database (CARD) (Alcock et al., 2023) protein homologue model. Optionally, sequences can also be annotated with AMRFinderPlus v4.0.19 (https://github.com/ncbi/amr) and Resfinder v4.4.2 (https://github.com/cadms/resfinder).

If binning is enabled, LRBinner v2.1 (https://github.com/anuradhawick/LRBinner) will bin reads in assembly-free mode, or COMEBin v1.0.3 (https://github.com/ziyewang/COMEBin) will bin contigs in assembly mode. Genome completeness and binning accuracy is then assessed with CheckM v1.2.3 (https://github.com/Ecogenomics/CheckM).

The deliverables from BALROG-MON are summaries that combine and/or visualize the pipeline results. The main output from BALROG-MON is the summary that creates a table based on the combination of Kraken2 sequence identities, Plasmer sequence classifications (Plasmid/Chromosome), and ARGs. This table allows the user to investigate each ARG and determine the putative origin or location (e.g., plasmid vs. chromosomal). Additionally, all quality control metrics, including those from raw sequence data, trimming, host/human sequence removal, and final read metrics, are summarized using MultiQC v1.22.3 (https://github.com/MultiQC/MultiQC). Lastly, if multiple ARG annotation tools are used, hAMRonization v1.0.2 (https://github.com/pha4ge/hAMRonization) standardizes outputs by harmonizing gene names, recording databases and software versions, and consolidating results into a single output. Additionally, an easy to use web-based report is generated that combines all samples for easy cross sample and cross database comparisons.

BALROG-MON is a robust and adaptable Nextflow pipeline developed for surveying pathogens and ARGs from metagenomic long-read sequencing data. By offering both "assembled" and "assembly-free" workflows, BALROG-MON overcomes the limitations of existing tools, providing researchers with a powerful solution to study ARGs in a wide range of samples, including methods to analyze host associated and low coverage metagenomes. The pipeline incorporates several key steps, including data quality control, read-based sequence identification, ARG and plasmid annotation, and optional metagenomic binning, ensuring a detailed and accessible analyses of complex metagenomic samples. Notable features include versatility in pipeline execution to handle difficult samples, the integration of multiple ARG databases, plasmid prediction capabilities, and optional binning for deeper analysis. BALROG-MON unifies these functionalities into a streamlined, user-friendly platform, simplifying the study of ARGs in metagenomic datasets. As the field of metagenomics advances, BALROG-MON stands poised to play a vital role in elucidating the dynamics of AMR and shaping strategies to address this global health challenge across human, animal, and environmental domains.
